# Factors associated with in-hospital mortality in acute exacerbations of COPD: a logistic regression and nomogram model study

**DOI:** 10.3389/fmed.2026.1789524

**Published:** 2026-05-07

**Authors:** Yang Liu, Yao Chen, Haoran Yang, Qinghua Wen, Jian Lv

**Affiliations:** 1 Department of Pharmacy, The People’s Hospital of Chongqing Liangping District, Chongqing, China; 2 Department of Pharmacy, Chongqing University Three Gorges Hospital, Chongqing, China

**Keywords:** acute exacerbation of chronic obstructive pulmonary disease, death outcome, influencing factors, logistic regression, nomogram model

## Abstract

**Objective:**

To explore the influencing factors of in-hospital mortality in patients with acute exacerbation of COPD (AECOPD).

**Materials and methods:**

Data of 425 patients with AECOPD were retrospectively collected and divided into a training set (*n* = 298) and a validation set (*n* = 127) at a ratio of 7:3. Risk factors for the death outcome were screened through univariate and multivariate logistic regression. A nomogram model was constructed, and the receiver operating characteristic curves (ROC) and calibration curves were plotted to evaluate the model’s performance, which was then validated in the validation set. Decision curve analysis (DCA) was used to evaluate the clinical value.

**Results:**

Multivariate logistic regression showed that age, blood oxygen saturation, alanine aminotransferase, and C-reactive protein were independent influencing factors for in-hospital mortality (*p* < 0.05). The importance ranking was C-reactive protein > age > oxygen saturation > alanine aminotransferase. The nomogram showed good calibration and discrimination in both the training and validation sets. The area under the ROC curve (AUC) was 0.871 (95% CI: 0.803–0.940) in the training set and 0.867 (95% CI: 0.778–0.956) in the validation set. The sensitivity and specificity were 0.912, 0.754 in the training set and 0.818, 0.771 in the validation set.

**Conclusion:**

The constructed model shows a promising association with in-hospital mortality in this single-center study, serving as a hypothesis-generating tool for risk stratification of patients with AECOPD.

## 1 Introduction

Chronic obstructive pulmonary disease (COPD), a chronic respiratory disease with high incidence, high disability rate, and high mortality worldwide. Its acute exacerbation has become a critical point leading to the deterioration of patients’ conditions and a sharp increase in the medical burden (1, 2). According to the latest clinical data, the short-term mortality rate of patients with acute exacerbation of COPD can reach 8–15%, and about 30% of the surviving patients will experience another acute exacerbation within 1 year, with a significant decline in their quality of life. During an acute exacerbation, patients often present with complex pathophysiological changes such as aggravated dyspnea, airway inflammatory storm, and involvement of multiple organ functions. The disease progresses rapidly and the prognosis varies significantly, posing great challenges to clinical diagnosis and treatment (3).

Currently, the clinical assessment of the outcomes of acute exacerbation of COPD mostly relies on empirical judgment, lacking objective and precise quantitative tools. Although previous studies have explored the associations between factors such as age, inflammatory indicators, and lung function and prognosis, most of these studies focused on single-factor analysis, and the conclusions were controversial (4, 5). For example, some studies considered the percentage of forced expiratory volume in one second (FEV1) to the predicted value as a key prognostic indicator, while others emphasized the importance of infection control. A systematic risk assessment system has not been established (6, 7). This difference in cognition makes it difficult for clinicians to implement early intervention for high-risk patients, missing the best treatment opportunity. In this context, this study innovatively integrates multi-dimensional clinical indicators, screens independent risk factors through a logistic regression model, and constructs a prediction model in combination with the nomogram visualization tool, aiming to develop a nomogram model to predict mortality in AECOPD, and rank the importance of predictors and validate the model. This study can not only fill the gap in current clinical risk assessment tools but also provide a quantitative basis for formulating individualized treatment strategies-for example, strengthening anti-inflammatory treatment for patients with high C-reactive protein levels and optimizing oxygen therapy regimens for patients with low oxygen saturation, ultimately achieving the goal of reducing mortality and improving patients’ quality of life.

## 2 Materials and methods

### 2.1 Study design and subjects

Clinical data of 425 patients with acute exacerbation of COPD who were treated in our hospital from January 2022 to December 2024 were collected. Inclusion criteria were as follows: (1) Meeting the diagnostic criteria for acute exacerbation of COPD in the “Guidelines for the Diagnosis and Treatment of Chronic Obstructive Pulmonary Disease (Revised Edition 2021)” (8); (2) Age ≥ 18 years; (3) Complete clinical data (including baseline data, laboratory tests, treatment, and prognosis information). For the four variables finally included in the model (age, SpO₂, ALT, CRP), there were no missing values. For other candidate variables, missing data were present in less than 5% of cases and were handled by complete-case analysis. Exclusion criteria were as follows: (1) Complicated with other serious pulmonary diseases such as lung cancer and pulmonary tuberculosis; (2) Complicated with severe organ failure of the heart, liver, kidneys, etc.; (3) Missing medical records or incomplete key data. The patients were randomly divided into a training set (*n* = 298) and a validation set (*n* = 127) at a ratio of 7:3 using the complete randomization method for model construction and validation (9).

### 2.2 Data collection

The following data of the patients were extracted through the electronic medical record system: (1) General information: gender, age, smoking history (≥10 packs/year), duration of COPD, history of acute exacerbation (number of attacks in the past 1 year), comorbidities (hypertension, diabetes, coronary heart disease); (2) Clinical indicators: pulmonary function grading (GOLD), percentage of FEV₁ to the predicted value, FEV₁/FVC ratio, oxygen saturation (SpO₂), medication use (inhaled corticosteroids, long-acting β₂-receptor agonists); (3) Laboratory tests: hemoglobin (HB), absolute neutrophil count (ANC), platelets (PLT), alanine aminotransferase (ALT), serum creatinine (Cr), total bilirubin (TBIL), C-reactive protein (CRP); (4) Treatment and prognosis: length of hospital stay, treatment outcome (death or survival). All laboratory tests and SpO₂ measurements were obtained within the first 2 h of admission. SpO₂ was measured using a pulse oximeter after the patient had been breathing room air without supplemental oxygen for at least 10 min. For patients who arrived on supplemental oxygen, a 10-min room air washout was performed if clinically safe; otherwise, the measurement was taken on the lowest feasible FiO₂ and noted.

### 2.3 Grouping criteria

Whether the patient died during hospitalization was regarded as the outcome event (dependent variable). Patients who died were included in the death group, and those who survived were included in the survival group.

### 2.4 Statistical analysis

Data analysis was performed using SPSS 26.0 and R 4.5.3. Categorical data were presented as the number of cases (percentage), and the chi-square test was used for comparison between groups. Measurement data conforming to the normal distribution were presented as the mean ± standard deviation, and the independent-samples *t*-test was used for comparison between groups. Initial variable selection was performed using Least Absolute Shrinkage and Selection Operator (LASSO) regression with 10-fold cross-validation to avoid overfitting and to handle potential multicollinearity. Variables with non-zero coefficients were then entered into multivariate logistic regression. Univariate logistic regression was also conducted for descriptive purposes, but final model selection was based on LASSO results. The nomogram model was constructed using the “rms” package in R language, and internal validation was carried out through the bootstrap method (1,000 repeated samplings). A calibration curve was drawn to evaluate the goodness-of-fit of the model, and the Hosmer-Lemeshow test was calculated to evaluate the model’s calibration. The receiver operating characteristic curve (ROC) was drawn, and the area under the curve (AUC), sensitivity, and specificity were calculated to evaluate the model’s discrimination. The clinical application value of the model was evaluated through decision curve analysis (DCA). To assess incremental value, we compared our model with the DECAF score using net reclassification improvement (NRI). Multicollinearity among the selected predictors was assessed using variance inflation factors (VIF).

## 3 Results

### 3.1 Comparison of baseline data of patients with Sjogren’s syndrome in the training set and the validation set

There were no statistically significant differences in baseline data such as gender, age, smoking history, duration of COPD, comorbidities, lung function indicators, medication use, and laboratory tests between the training set and the validation set (*p* > 0.05), indicating comparability (Table 1).

**Table 1:** Comparison of general clinical characteristics of patients in the training set and the validation set.

Indicators	Training set (*n* = 298)	Validation set (*n* = 127)	*χ* ^2^ */t*	*P*
Sex (Male/Female)	211/87 (70.81/29.19)	92/35 (72.44/27.56)	0.116	0.733
Age (years)	66.94 ± 8.59	67.85 ± 9.21	0.978	0.329
Smoking history (Yes/No)	225/73 (75.51/24.49)	95/32 (74.81/25.19)	0.024	0.878
COPD duration (years)	8.95 ± 4.56	9.21 ± 5.12	0.518	0.605
History of acute exacerbation (in the past 1 year, 0 times/1 time and above)	56/242 (18.79/81.21)	34/93 (26.77/73.23)	3.397	0.065
Comorbid hypertension (Yes/No)	148/150 (49.66/50.34)	60/67 (47.24/52.76)	0.209	0.648
Comorbid diabetes (Yes/No)	35/263 (11.74/88.26)	17/110 (13.39/86.61)	0.223	0.637
Comorbid coronary heart disease (Yes/No)	76/222 (25.51/74.49)	34/93 (26.77/73.23)	0.075	0.785
Pulmonary function grading (GOLD) (Grade I–II/Grade III–IV)	162/136 (54.36/45.64)	67/60 (52.76/47.24)	0.093	0.761
Percentage of FEV1 to predicted value (%)	35.04 ± 5.74	36.12 ± 5.88	1.763	0.079
FEV1/FVC ratio (%)	46.12 ± 4.93	46.86 ± 4.32	1.468	0.143
Oxygen saturation (SpO₂) (%)	88.61 ± 6.05	87.65 ± 6.12	1.492	0.136
Inhaled corticosteroids (ICS, Yes/No)	184/114 (61.74/38.26)	87/40 (68.51/31.49)	1.761	0.185
Long-acting β₂-receptor agonist (LABA, Yes/No)	181/117 (60.74/39.26)	85/42 (66.93/33.07)	1.458	0.227
Long-acting anticholinergic drug (LAMA, Yes/No)	176/122 (59.06/40.94)	83/44 (65.35/34.65)	1.482	0.224
Administration route of doxofylline (Intravenous/Oral)	159/139 (53.35/46.65)	77/50 (60.63/39.37)	1.908	0.167
Medication compliance (Compliant/Non-compliant)	92/206 (30.87/69.13)	35/92 (27.56/72.44)	0.467	0.495
Hemoglobin (HB) (g/L)	120.03 ± 15.28	122.52 ± 16.23	1.509	0.132
Absolute neutrophil count (ANC) (×10 ^9^ /L)	9.94 ± 2.58	9.86 ± 3.12	0.274	0.784
Platelets (PLT) (×10^9^/L)	231.71 ± 55.06	223.52 ± 56.52	1.393	0.165
Alanine aminotransferase (ALT) (U/L)	35.78 ± 12.02	37.82 ± 13.52	1.542	0.124
Serum creatinine (Cr) (μmol/L)	96.25 ± 12.81	98.65 ± 13.25	1.749	0.081
Total bilirubin (TBIL) (μmol/L)	16.81 ± 6.32	17.12 ± 6.53	0.458	0.647
C-reactive protein (CRP) (mg/L)	47.16 ± 21.21	49.26 ± 18.26	0.9730	0.331

### 3.2 Univariate analysis of the death outcome of patients with acute exacerbation of chronic obstructive pulmonary disease in the training set

In the training set, 28 patients (9.39%) died and 270 patients (90.61%) survived. Univariate analysis showed that there were statistically significant differences in age, blood oxygen saturation, alanine aminotransferase (ALT), C-reactive protein (CRP), and length of hospital stay between the death group and the survival group (*p* < 0.05), while there were no statistically significant differences in indicators such as gender, smoking history, and comorbidities (*p* > 0.05) (Table 2).

**Table 2:** Univariate analysis of the death outcome of patients with acute exacerbation of COPD in the training set.

Indicators	Death group (*n* = 28)	Survival group (*n* = 270)	*χ* ^2^ */t*	*P*
Sex (Male/Female)	22/6 (78.57/21.43)	189/81 (70.00/30.00)	0.902	0.342
Age (years)	72.35 ± 7.15	66.39 ± 8.56	3.556	0.001
Smoking history (Yes/No)	25/3 (89.29/10.71)	200/70 (74.07/25.93)	3.174	0.075
COPD duration (years)	10.12 ± 4.32	8.83 ± 4.58	1.426	0.155
History of acute exacerbation (in the past 1 year, 0 times/1 time and above)	2/26 (7.14/92.86)	54/216 (20.00/80.00)	2.748	0.097
Comorbid hypertension (Yes/No)	15/13 (53.57/46.43)	133/137 (49.26/50.74)	0.188	0.664
Comorbid diabetes (Yes/No)	5/23 (17.86/82.14)	30/240 (11.11/88.89)	0.558	0.455
Comorbid coronary heart disease (Yes/No)	11/17 (39.29/60.71)	65/205 (24.07/75.93)	3.537	0.061
Pulmonary function grading (GOLD) (Grade I–II/Grade III–IV)	12/16 (42.86/57.14)	150/120 (55.56/44.44)	1.649	0.199
Percentage of FEV1 to predicted value (%)	33.12 ± 6.35	35.25 ± 5.65	1.876	0.062
FEV1/FVC ratio (%)	44.85 ± 5.26	46.35 ± 4.89	1.534	0.126
Oxygen saturation (SpO₂) (%)	85.36 ± 6.26	88.95 ± 5.95	3.024	0.003
Inhaled corticosteroids (ICS, Yes/No)	22/6 (78.57/21.43)	162/108 (59.26/40.74)	3.704	0.054
Long-acting β₂-receptor agonist (LABA, Yes/No)	22/6 (78.57/21.43)	169/101 (62.59/37.41)	2.815	0.093
Long-acting anticholinergic drug (LAMA, Yes/No)	21/7 (75.00/25.00)	155/115 (57.41/42.59)	3.247	0.072
Administration route of doxofylline (Intravenous/Oral)	19/9 (67.86/32.14)	140/130 (51.85/48.15)	2.611	0.106
Medication compliance (Compliant/Non-compliant)	11/17 (39.29/60.71)	81/189 (30.00/70.00)	1.025	0.311
Hemoglobin (HB) (g/L)	115.89 ± 16.15	120.89 ± 15.15	1.652	0.099
Absolute neutrophil count (ANC) (×10^9^/L)	10.85 ± 2.63	9.85 ± 2.56	1.963	0.051
Platelets (PLT) (×10^9^/L)	225.62 ± 58.36	235.66 ± 55.52	0.907	0.365
Alanine aminotransferase (ALT) (U/L)	41.26 ± 12.85	35.22 ± 11.82	2.806	0.005
Serum creatinine (Cr) (μmol/L)	100.35 ± 11.84	95.82 ± 12.85	1.788	0.075
Total bilirubin (TBIL) (μmol/L)	18.65 ± 6.22	16.62 ± 6.32	1.621	0.106
C-reactive protein (CRP) (mg/L)	65.28 ± 21.33	45.28 ± 20.36	4.926	0.001

### 3.3 Multivariate logistic regression analysis of treatment outcomes in the acute phase of chronic obstructive pulmonary disease

Multivariate logistic regression showed that age (OR = 1.114, 95%CI: 1.042–1.191, *p* = 0.001), oxygen saturation (OR = 0.883, 95%CI: 0.814–0.958, *p* = 0.003), alanine aminotransferase (ALT) (OR = 1.040, 95%CI: 1.004–1.077, *p* = 0.030), and C-reactive protein (CRP) (OR = 1.049, 95%CI: 1.025–1.074, *p* = 0.001) were independent influencing factors for in-hospital mortality (Table 3). The complete logistic regression equation for predicting in-hospital death probability (*P*) was: logit(*P*) = −5.872 + 0.108 × Age − 0.124 × SpO₂ + 0.039 × ALT + 0.048 × CRP. All VIF values were below 2, confirming no significant multicollinearity.

**Table 3:** Multivariate logistic regression analysis.

Factor	β	SE	Wald	*P*	OR	95%CI
Age	0.108	0.034	10.105	0.001	1.114	1.042–1.191
Oxygen saturation	−0.124	0.042	8.712	0.003	0.883	0.814–0.958
Alanine aminotransferase	0.039	0.018	4.694	0.030	1.040	1.004–1.077
C-reactive protein	0.108	0.034	10.105	0.001	1.114	1.042–1.191

### 3.4 Importance ranking of influencing factors

**Figure 1:**
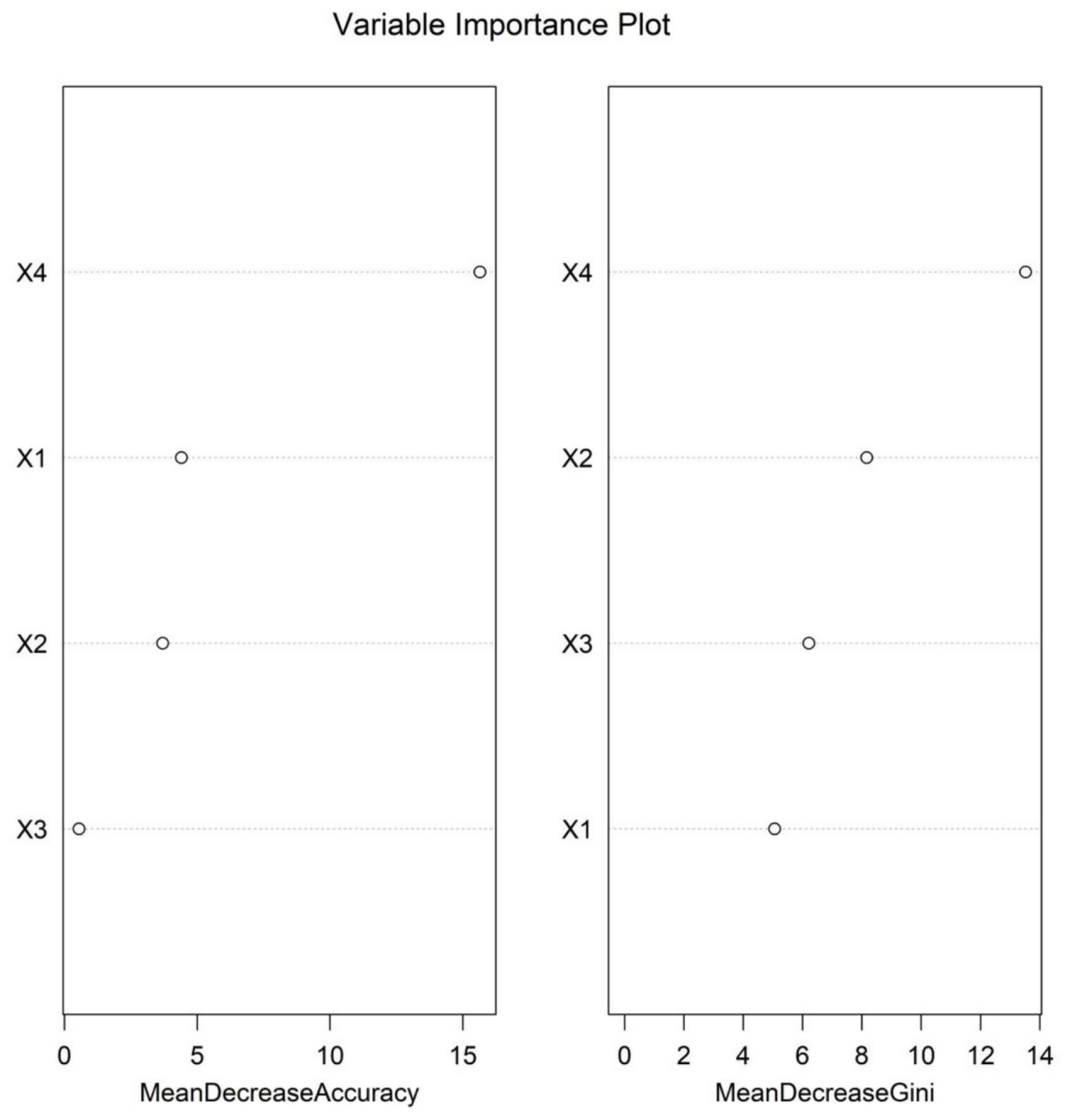
Importance ranking of influencing factors. x1: Age; x2: Oxygen saturation; x3: Alanine aminotransferase; x4: C-reactive protein.

Based on the absolute values of the standardized regression coefficients of each influencing factor obtained from the multivariate logistic regression analysis, the importance of the influencing factors was ranked (the variable assignment was shown in Table 4). The result was CRP > age > oxygen saturation > ALT (Figure 1).

**Table 4:** Variable assignment methods.

Variable	Meaning	Assignment
x1	Age	Continuous variable
x2	Oxygen saturation	Continuous variable
x3	Alanine aminotransferase	Continuous variable
x4	C-reactive protein	Continuous variable
Y	Death	No = 0, Yes = 1

### 3.5 Establishment of the nomogram prediction model

**Figure 2:**
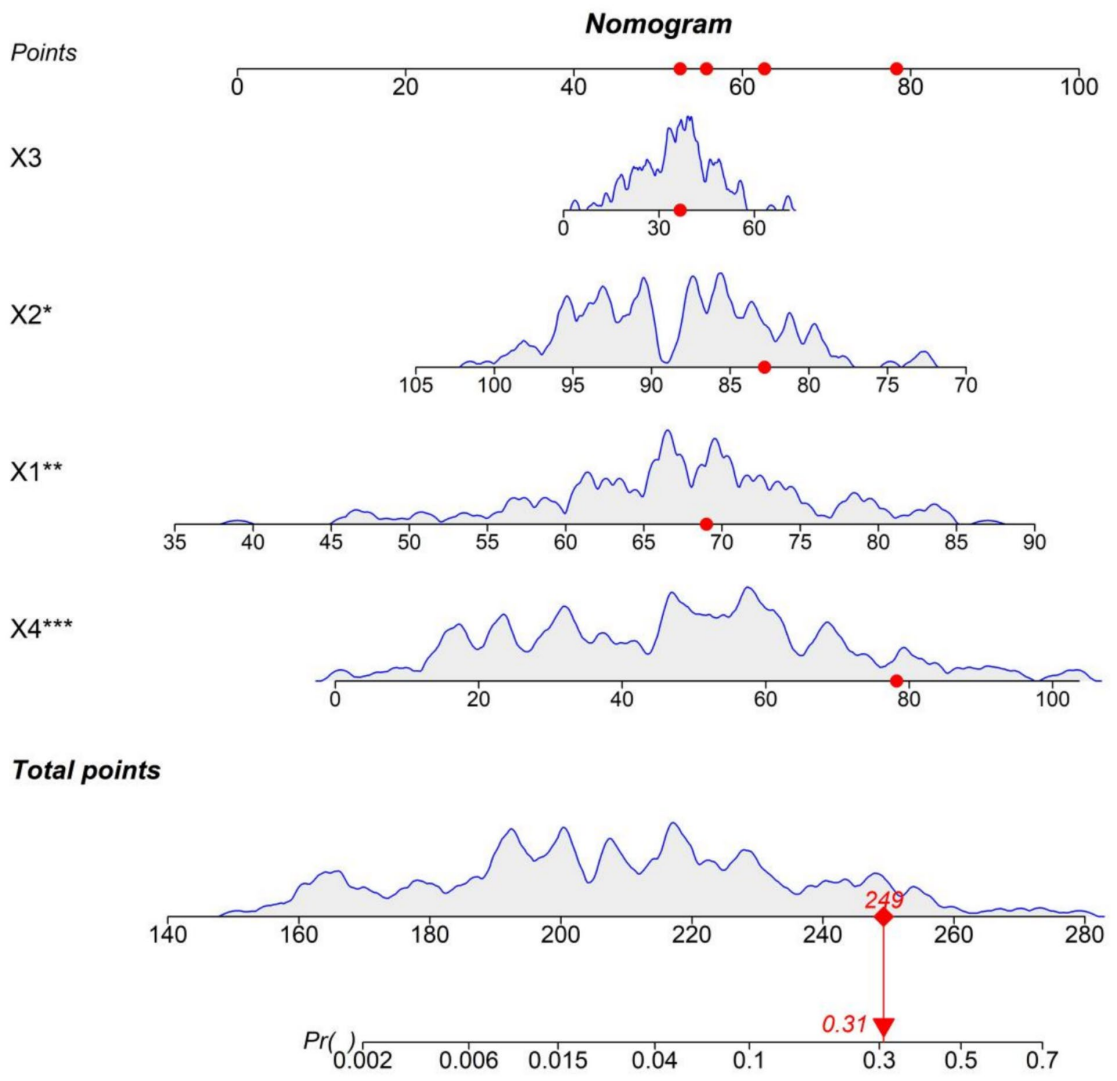
Nomogram model for the death outcome of patients with acute exacerbation of chronic obstructive pulmonary disease (AECOPD). x1: Age; x2: Oxygen saturation; x3: Alanine aminotransferase; x4: C-reactive protein.

Based on the results of the multivariate logistic regression analysis, a nomogram model for predicting the death outcome of patients with acute exacerbation of COPD was constructed. Scores were assigned to each independent risk factor, and the total score was obtained by summing up the scores of each factor. The predicted probability of the death outcome could be obtained from the nomogram model through the total score (Figure 2).

### 3.6 Evaluation and validation of the nomogram model for the death outcome of patients with AECOPD

**Figure 3:**
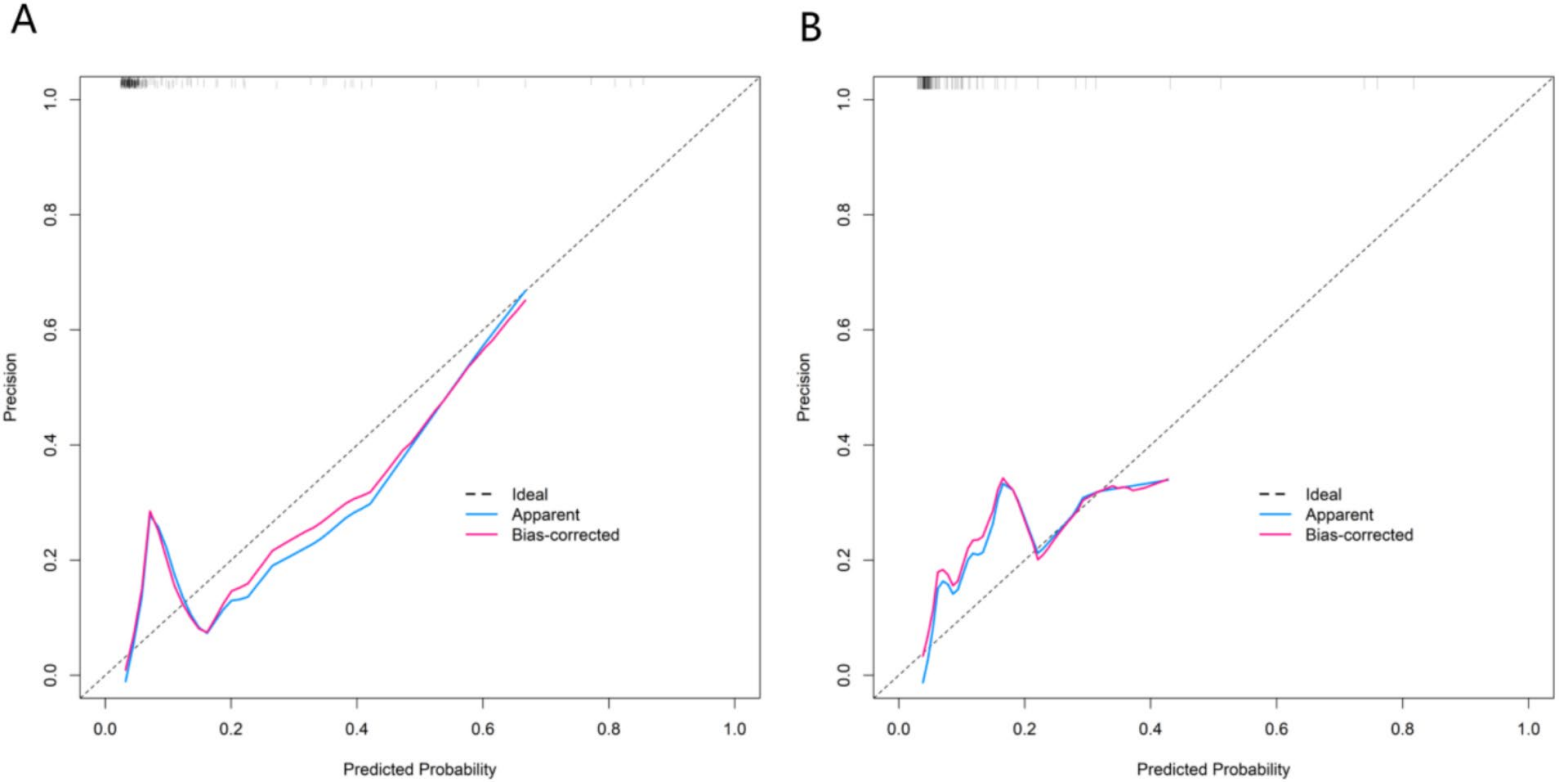
Calibration curves in the training set **(A)** and the validation set **(B)**.

**Figure 4:**
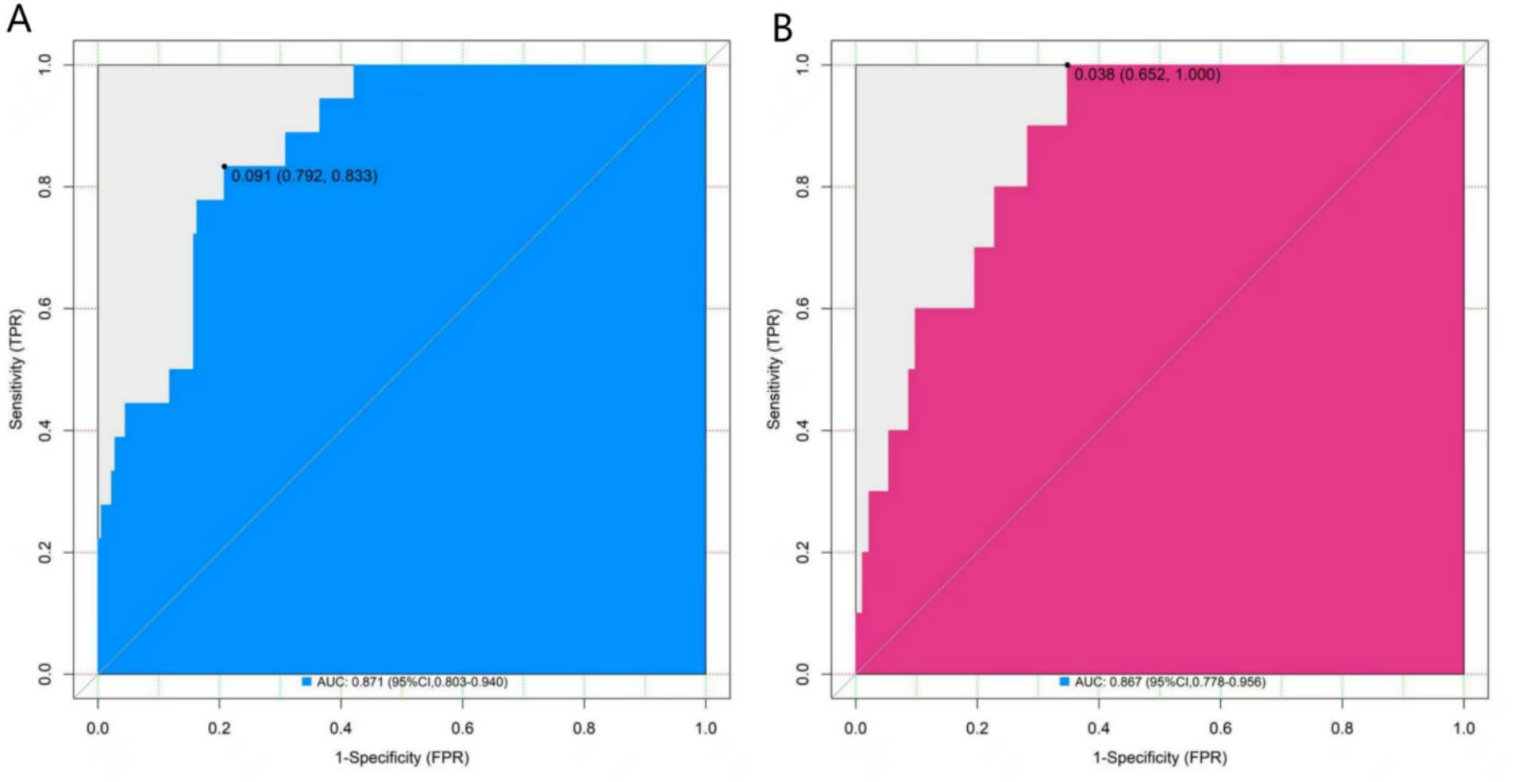
ROC curves in the training set **(A)** and the validation set **(B)**.

In the training set, the Hosmer-Lemeshow test gave a *p* value of 0.602, indicating good calibration. The ROC curve showed an AUC of 0.871 (95% CI: 0.803–0.940), with a sensitivity of 0.912 and a specificity of 0.754. In the validation set, the Hosmer-Lemeshow test gave a p value of 0.784, and the AUC was 0.867 (95% CI: 0.778–0.956), with a sensitivity of 0.818 and a specificity of 0.771. Quantitative calibration metrics: calibration slope = 0.91 (ideal = 1.00), calibration in the large = −0.06 (ideal = 0). Comparison with the DECAF score: In the training set, the DECAF score achieved an AUC of 0.742 (95% CI: 0.651–0.833), which was lower than our nomogram’s AUC (0.871, *p* = 0.03). The NRI for our model compared to DECAF was 0.21 (95% CI: 0.01–0.42, *p* = 0.04). These comparisons are exploratory due to the limited sample size. The calibration curves and the ROC curves were shown in Figures 3, 4.

### 3.7 Decision curve analysis

**Figure 5:**
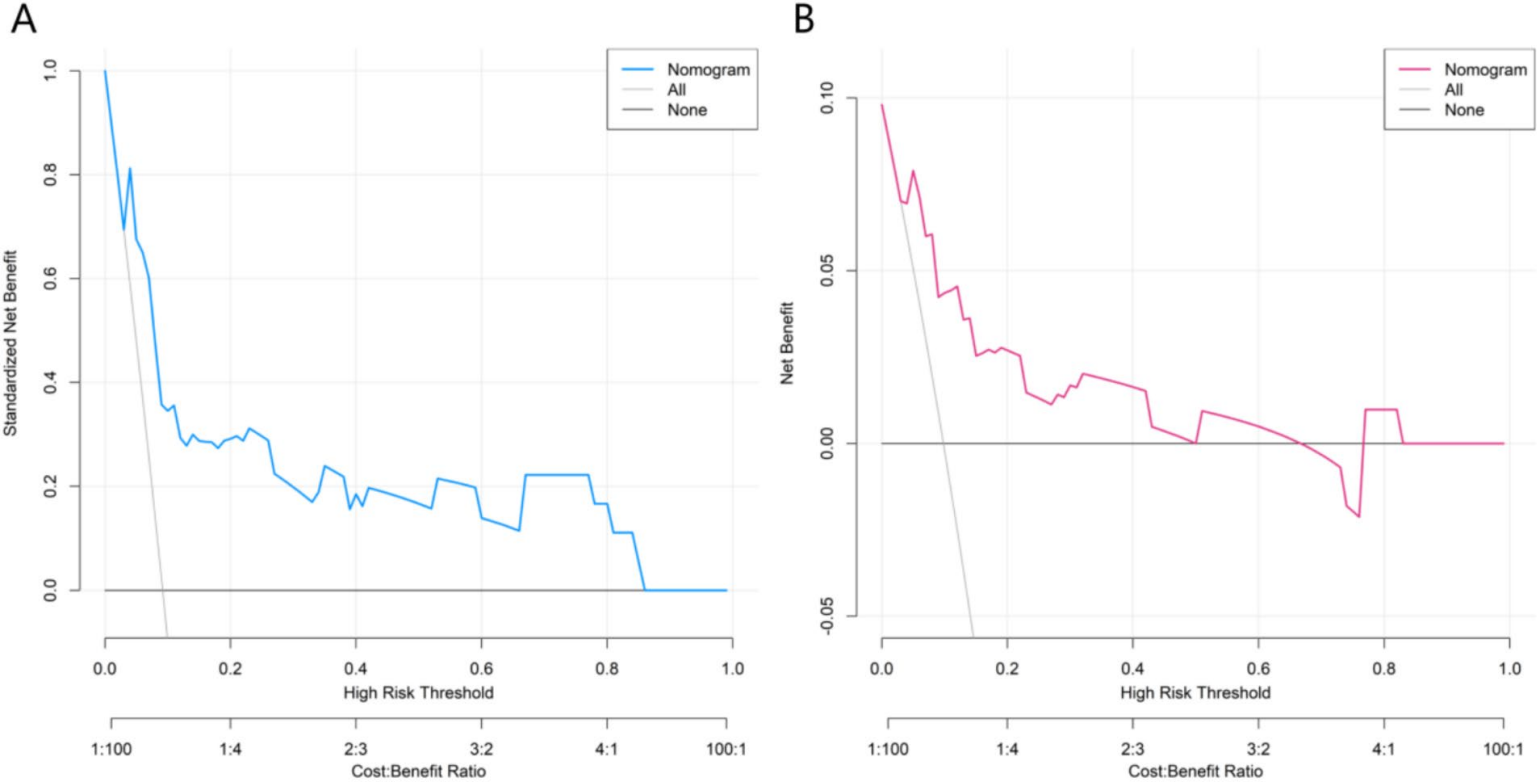
Decision curves in the training set **(A)** and the validation set **(B)**.

DCA showed that when the threshold probability was between 0.06 and 0.80, the net benefit of the nomogram was higher than that of the “treat-all” or “treat-none” strategies, suggesting clinical usefulness (Figure 5).

## 4 Discussion

In this study, through a retrospective analysis of 425 AECOPD patients, logistic regression identified four independent factors associated with in-hospital mortality: age, SpO₂, ALT, and CRP. A nomogram was constructed based on these admission-available variables. The mortality rates were 9.39% in the training set and 8.66% in the validation set. The AUCs were 0.871 in the training set and 0.867 in the validation set. Calibration was satisfactory (slope 0.91, CITL −0.06). The calibration curve indicated a good consistency between the predicted values and the actual values, and the decision curve analysis confirmed its significant clinical application value. This result not only identified the core driving factors of the death risk in patients in the acute phase of COPD but also provided a visual and quantifiable risk assessment tool, laying a foundation for precise clinical intervention.

In this study, potential risk factors were first screened from more than 20 clinical indicators using LASSO regression, and four independent influencing factors (age, SpO₂, ALT, CRP) were finally identified through multivariate analysis. This process effectively excluded confounding factors and ensured reliability. Multivariate regression showed that all four indicators were statistically significant (*p* < 0.05). CRP had the highest importance ranking (based on standardized coefficients), while age had the same OR (1.114). The step-by-step screening method retained key information and avoided overfitting, providing a robust foundation for model construction. The nomogram transformed the complex regression equation into an intuitive visual tool by assigning scores and summing them up. Clinicians could quickly obtain the probability of in-hospital mortality using the patient’s indicators, improving practicality. Calibration curves showed good agreement between predicted and observed risks, and the Hosmer-Lemeshow test *p* values were >0.05 in both sets (0.602 and 0.784), confirming good calibration. In ROC analysis, the AUC values were 0.871 (95%CI:0.803–0.940) and 0.867 (95%CI:0.778–0.956), with narrow confidence intervals suggesting high stability. The sensitivity and specificity were 0.912 and 0.754 in the training set and 0.818 and 0.771 in the validation set, indicating that the model could effectively identify high-risk patients while reducing over-diagnosis. Decision curve analysis showed that when the threshold probability was between 0.06 and 0.80, the net benefit of the nomogram was higher than that of the “treat-all” or “treat-none” strategies, suggesting that the model could guide clinical decision-making and help balance treatment risks and benefits.

In this study, CRP ranked first in terms of importance (OR = 1.114), and an increase in its level was significantly associated with the death risk. As a classic inflammatory marker, CRP can reflect the severity of airway inflammation in the acute phase of COPD. Studies have shown that during acute exacerbations of COPD, neutrophil infiltration in the airways and cytokine storms (such as IL-6, TNF-α) can lead to a sharp increase in CRP levels, and a high-inflammation state can exacerbate lung tissue damage, induce systemic inflammatory response syndrome (SIRS), and even lead to multiple organ dysfunction. In this study, the mean CRP level in the death group (65.28 mg/L) was significantly higher than that in the survival group (45.28 mg/L), confirming that uncontrolled inflammation is the core mechanism of poor prognosis (10, 11). This finding suggested that dynamic monitoring of CRP levels in clinical practice can provide an important basis for assessing the disease progression. Early intensive anti-inflammatory treatment (such as glucocorticoid pulse therapy and targeted biological agents) for patients with high CRP levels may improve the prognosis (12–14).

Blood oxygen saturation is a direct indicator of the body’s oxygenation status. In this study, it ranked third in importance after CRP (OR = 0.883), after CRP and age. Lower oxygen saturation at admission is associated with higher mortality. Patients in the acute phase of COPD often have hypoxemia due to airway obstruction and ventilation-perfusion mismatch, and continuous hypoxia can lead to a series of reactions such as myocardial hypoxia, brain dysfunction, and renal function damage. Studies have shown that SpO₂ < 88% is a strong predictor of short-term death in COPD patients, which is consistent with the result in this study that the mean SpO₂ in the death group (85.36%) was significantly lower than that in the survival group (88.95%) (15–17). This suggested that maintaining blood oxygen saturation within the target range (usually ≥90%) may be a key measure to improve the prognosis. In clinical practice, the oxygen therapy plan should be optimized as early as possible, and non-invasive or invasive ventilation support should be used if necessary.

An increase in age is an important risk factor for death in the acute phase of COPD (OR = 1.114). For every one-year increase in age, the death risk increases by 11.4%. Elderly patients often have a decline in lung function reserve, a decline in immune function, and multiple underlying diseases (such as hypertension and diabetes), and their tolerance to acute exacerbations is significantly reduced (18, 19). In this study, the average age of the death group was higher than that of the survival group, which was consistent with the conclusion in previous studies that “age > 70 years is an independent predictor of short-term death”. This suggested that more active preventive measures (such as vaccination and optimization of the management of underlying diseases) should be taken for elderly patients, and intensive care should be initiated earlier during acute exacerbations (20, 21).

An increase in ALT (OR = 1.040) indicates liver function damage, which in the context of AECOPD may also be a marker of systemic congestion from right heart failure or sepsis-related hepatopathy, as discussed in GOLD 2025/2026 guidelines. Although it ranked lowest in importance in this study, it was still an independent factor associated with in-hospital mortality. The systemic inflammatory response, hypoxia, and drug toxicity (such as certain antibiotics and hormones) in the acute phase of COPD can all lead to liver cell damage, and abnormal liver function can further affect metabolism, immunity, and coagulation function, forming a vicious circle. Studies have shown that an increase in ALT in COPD patients is significantly associated with the 30-day mortality. The results of this study support this view, suggesting that in clinical practice, liver function monitoring should be paid attention to, hepatotoxic drugs should be avoided, and liver-protecting treatment should be given if necessary (22, 23).

Compared with previous single-factor studies, this study screened out four core indicators through logistic regression and first clarified their importance ranking, providing a multi-dimensional risk assessment framework of “inflammation-oxygenation-disease course-age-liver function” for clinical practice. The nomogram model quantifies and assigns scores to each indicator, transforming the abstract risk assessment into an intuitive numerical probability, which is convenient for rapid clinical application. In this study, the training set and the validation set were split (7:3), and internal validation was carried out through the bootstrap method to ensure the stability of the model. The DCA analysis directly confirmed the net benefit advantage of the model within a wide range of thresholds, making up for the limitations of a simple statistical efficacy evaluation and providing a solid basis for clinical promotion.

Some limitations in this study should be considered. First, our model does not include blood eosinophil counts, a key biomarker recommended by the GOLD 2025/2026 guidelines, because these data were not routinely collected in our electronic medical records during the study period. Second, although we included baseline FEV1% and GOLD grade, they were not selected as independent predictors, possibly due to collinearity with other variables or limited sample size. Third, the interpretation of ALT elevation should consider extrapulmonary comorbidities such as right-sided heart failure, as noted in GOLD Chapter 5. Fourth, the events-per-variable ratio in the training set was 7.0 (28 events/4 predictors), below the recommended threshold of 10, increasing the risk of overfitting. Therefore, our findings should be considered hypothesis-generating. Fifth, as a single-center study with only temporal internal validation, external validation in independent multicenter study is required before any clinical application. Sixth, the follow-up time was limited to in-hospital outcomes. Future studies should include longer follow-up and more diverse populations.

In conclusion, the nomogram constructed in this study identifies four factors (age, SpO₂, ALT, CRP) associated with in-hospital mortality in AECOPD. The model shows promising discrimination and calibration in this single-center study, but it should be used only as a hypothesis-generating tool. External validation in larger, multicenter study is essential before any clinical implementation.

## Data Availability

The original contributions presented in the study are included in the article/supplementary material, further inquiries can be directed to the corresponding author.
